# Innovations for the elimination and control of visceral leishmaniasis

**DOI:** 10.1371/journal.pntd.0007616

**Published:** 2019-09-19

**Authors:** Angamuthu Selvapandiyan, Simon L. Croft, Suman Rijal, Hira L. Nakhasi, Nirmal K. Ganguly

**Affiliations:** 1 Institute of Molecular Medicine, Jamia Hamdard, New Delhi, India; 2 Faculty of Infectious and Tropical Diseases, London School of Hygiene and Tropical Medicine, London, United Kingdom; 3 Drugs for Neglected Diseases Initiative, New Delhi, India; 4 Division of Emerging and Transfusion Transmitted Diseases, Center for Biologics Evaluation and Research, Food and Drug Administration, Silver Spring, Maryland, United States of America; 5 Apollo Hospitals Educational and Research Foundation, New Delhi, India; Ohio State University, UNITED STATES

Visceral leishmaniasis (VL), a disease associated with poverty, is endemic in the Indian subcontinent and Africa (where it is caused by the protozoan parasite *Leishmania donovani*, and in Latin America and the Mediterranean region (where it is caused by *L*. *infantum*). In all regions, it is transmitted by the female sand fly vector. Although there has been a substantial decline in the number of reported cases in recent years, VL continues to affect many tropical and subtropical countries, despite international, national, and local efforts towards its control and elimination over the past several decades. In 2005, a target for the elimination of VL, defined as reducing incidence to a level where it would no longer be of public health importance, i.e., <1 per 10,000 inhabitants per year at each health intervention unit from India, Nepal, and Bangladesh, was set for 2015. This target date was missed, as was a second target date of 2017. WHO has recently reset the target date to 2020 for the elimination of this disease from the Indian subcontinent (ISC).

To review the progress and prospects made towards elimination, an International Conference on Innovations for the Elimination and Control of Visceral Leishmaniasis (IEC-VL’18) was held in New Delhi, India from 28–30 November 2018. Discussions and debates throughout the conference were aimed to provide a focus for stakeholders and decision makers to frame further control measures and policies and to define needed research and tools. Thus, innovations that would be key during the “last mile” towards reaching the elimination targets needed emphasis. A focus on the challenges due to loss of immunity, parasite and human population heterogeneities, noncompliance to control measures, and reasons for outbreaks and resurgence of the disease is needed. Significant advances in the discovery and development of new drugs, diagnostics, vaccines, and vector control measures that offer opportunities for future interventions need to be highlighted.

## Progress made during the elimination program

From 2005, the year of regional elimination initiative in Nepal, there has been decline in the kala-azar (VL) cases from 2,220/10,000 in 2003 to only 254 in 2017. However, in recent years, the region has witnessed an increase in the number of cases coming from previously nonendemic hilly regions [1,2]. The cases of untreated post-kala-azar dermal leishmaniasis (PKDL) patients, a sequela of VL after treatment, has also increased; this potential reservoir of *Leishmania* creates concerns over the possibility of future epidemics [2]. Recent data, based upon sand fly feeding studies, suggests that disease transmission from PKDL cases is likely, with feeding on nodular PKDL being more likely to result in positive xenodiagnoses than macular PKDL [3,4]. In the ISC, 10%–15% of reportedly cured cases of VL develop PKDL. Modeling of cases reported indicates that achievement of elimination in low VL endemic areas will be difficult [5,6]. Apart from PKDL as a reservoir, there are estimates of 5%–10% asymptomatic to symptomatic conversions per annum, adding a challenge to the elimination process [7,8]; the emergence of HIV-VL coinfection is another challenge [9].

## Issues with diagnosis

The rK39 dipstick test has been an essential diagnostic tool for VL over the past decade. However, as this test has variable sensitivity and limited use for PKDL and VL-HIV cases, novel tools are required. Overcoming the limitations of these diagnostic tests, further development and approval of nucleic acid amplification methods, such as the loop-mediated isothermal amplification (LAMP) assay [10,11] and urine antigen detection using ELISA [12,13], are in the pipeline to provide a rapid and reliable diagnosis of VL and PKDL, capable of assessing the cure and monitoring the efficacy of new antileishmanial drugs. Adding additional challenge for sustained elimination, we also need a noninvasive antigen-based test that can be used for surveillance.

## Drug treatment

Over the past decade, the use of oral miltefosine and single-course AmBisome (liposomal amphotericin B) since 2014 for VL have been the basis for the elimination program, although a comprehensive evaluation in their role in reducing patient numbers has not been made. Both treatments have limitations, with a 28-day regime and side effects, along with reported increase in miltefosine treatment failure [14] and the need for a cold-chain for AmBisome making additional demands, which have been supported by KalaCORE (a partnership to support the control and elimination of VL) and WHO. There also need to be follow-up studies to show whether the single course AmBisome regime is associated with higher PKDL incidence. These limitations have provided the rational for essential research on combination therapy and the identification of novel classes of chemical entities (e.g., oxoboroles, aminopyrazoles, and nitroimidazoles [15]. Currently efficacious combinations, using existing drugs, of miltefosine-paromomycin or miltefosine-AmBisome are recommended in the region for VL. Combinations also offer hope for co-infections; in Ethiopia, AmBisome plus miltefosine have proved efficacious in HIV-VL patients [16]. Host-related therapies (for example, liposomal cholesterol) that have been effective in treatment of experimental VL model [17] deserve to be considered. To support studies on new treatments, we need much-improved serological, immunological, and genetic markers for clinical use that can determine progression from *L*. *donovani* infection to clinical VL. Markers for asymptomatic infections have been used in clinical studies [18,19]. However, in the absence of specific safe drugs or markers of disease progression, studies on how to deal with these infections are needed.

## Prevention using vaccination

With all the issues and adversities related to *Leishmania* treatment and management, prevention remains the key to sustainable elimination. Several vaccine strategies have been pursued using recombinant peptide, DNA, killed whole parasite and genetically modified live-attenuated parasites [20,21]. Among them, *L*. *donovani* centrin gene knockout parasite has been reported to generate strong protective immune response in the animals against VL [22,23]. Another promising centrin-deleted *L*. *major* vaccine candidate is currently being developed using marker-free CRISPR Cas9 technology. These parasites are safe and generate potent innate and adaptive protective immune response against both CL and VL. Currently, this vaccine candidate is being tested in dogs in endemic region and manufactured under good laboratory practice (GLP) and good manufacturing practice (GMP) conditions for future clinical trials. DNA and therapeutic vaccines are being actively developed by other laboratories. ChAd63-KH, an adenoviral vaccine encoding KMP-11 and HASPB, is currently in Phase II as a therapeutic vaccine for persistent PKDL in Sudan [24]. In addition, the new Phase IIa/b trial to assess whether ChAd63-KH as vaccine can avert the progression of VL to PKDL in previously treated VL patients will begin in Sudan soon. The process of technology transfer for manufacturing F3+/GLA-SE vaccine **(**LEISH-F3 recombinant protein antigen formulated with GLA-SE) against VL by Infectious Research Institute, Seattle, WA, USA after extensive antigen discovery and adjuvant development is in process [5]. Further preclinical development of a 5-antigen DNA vaccine [25] also continues.

## Prevention through sand fly control

Although the elimination target in the ISC is currently defined as a public health problem, the accepted definitions of elimination [26,27] are based upon the interruption of transmission. However, we do not have a proper measure of transmission of VL. As per a study in Bihar, no correlation was found between the presence of infected sand flies and the clinical VL occurrence [28]. Hence, further research is needed. Nevertheless, there are reports predicting the infected sand fly clusters to correlate to the disease burden in Nepal and Spain [29,30]. The gaps in vector bionomics knowledge about leishmaniasis elimination include estimates of sand fly biting rates, parasite infection rates in the vector, and the spatial and temporal variations of these in response to indoor residual spraying (IRS) [31]. Addressing such knowledge gaps are of paramount importance to achieve sustained VL elimination within a policy focused on an appropriate post elimination program. The data on the poorly understood dispersal of the vector needs to be collected for better IRS. Because the sand fly modified its mostly endophilic (living indoors) habitat from past to exophilic (life outdoors) now [32], special tools targeting the vector with the exophilic habitat may be needed along with IRS.

Further to the outcomes of this discussion, we invite submission of articles or reviews to *PLOS Neglected Tropical Diseases* on the following:

1. Papers that elaborate on the innovations related to elimination and control of leishmaniasis including VL, cutaneous leishmaniasis, and post-kala-azar leishmaniasis in the areas of current status, genomics and basic science, immunology, epidemiology, transmission, diagnostics, drugs, vaccines and data, and health systems.
2. Papers on vector control measures handled to control sand fly population to reduce transmission.

Through the suggestions and kind support of various stakeholders, we will continue in our efforts towards the sustained elimination of VL from the ISC and improved control in East Africa as a goal by continued networking, conducting meetings, monitoring progress, updating information via publications, including publications in *PLOS Neglected Tropical Diseases*, until we have the tools, the health system structure, and funding to ensure elimination of VL based upon sustainable interruption of transmission.

## Disclaimer

Contributions by Dr. Nakhasi are an informal communication and represent his own best judgment. These comments do not bind or obligate the United States Food and Drug Administration.
